# Alzheimer's Patient Organizations' Role in Enabling Citizenship Projects: A Comparison of the USA, Germany, and the UK

**DOI:** 10.3389/fsoc.2020.00019

**Published:** 2020-04-02

**Authors:** Sayani Mitra, Silke Schicktanz

**Affiliations:** Department for Medical Ethics and History of Medicine, University Medical Center Göttingen, Göttingen, Germany

**Keywords:** patient organizations, Alzheimer's disease, citizenship, rights, comparative research

## Abstract

This paper examines how Alzheimer's disease (AD) patient support organizations (POs) located in diverse healthcare regimes enable patients to claim and construct their rights as citizens. Since citizenship rights of people with AD are debated widely, it is important to recognize the role of POs in enabling people to construct citizenship identities. This paper thus examines the factors that shape the citizenship projects of the AD POs. Since collective health-related behavior changes in line with national differences, we compare the biggest AD POs in three starkly distinct healthcare regimes: the Alzheimer's Association in the US (ALZ), the Deutsche Alzheimer Gesellschaft (German Alzheimer's Association) in Germany (DAG), and Alzheimer's Society in the UK (AS), to examine how distinct health policy contexts shape their citizenship projects. Based on our website analysis of the three POs and other related secondary documents, we argue that the way each POs work toward enabling its members to claim rights and assume responsibilities depend upon the nature of healthcare funding and resource allocation for AD care. Since AD involves long-term care, the ways in which the three POs enable the people with AD to secure their care expenses set apart the nature of citizenships enactments.

## Introduction

Dementia, including Alzheimer's disease (AD) is considered as one of the leading healthcare problems around the world. Patient support organizations (POs) have been playing a cardinal role in addressing this crucial healthcare crisis through their activities of care, communication, and advocacy. But often such engagement remains limited to representation of issues as an act of upholding patients' epistemic authorities rather than concretely engaging in the decision-makings of policies that impact them (Jongsma et al., 2017; Madden and Speed, 2017). Yet POs enable the patients to identify their rights and understand their responsibilities. These often take the form of citizenship projects as they create arenas for its members to perform their identities and claim their entitlements.

Citizenship is concerned about rights and responsibilities of individuals as well as the state (Oliver and Heater, 1994; Bartlett and O'Connor, 2007). By promoting inclusionary practices in the designing of the healthcare benefits (e.g., by joining national health committees or giving advice to politicians) POs enable people, especially the vulnerable ones to exercise the terms of their citizenship (Barnes and Brannelly, 2008). Although the AD movement was initiated by, and originally intended for the carers rather than the people with AD (Beard, 2004), many AD POs at present publicly identify themselves as carers' *and* patients' organizations, reflecting a process of hybridization of their claims and recognition of those affected (O'Donovan et al., 2013; Schicktanz et al., 2018). Due to this hybridity of representation and questioning of political citizenships of people with AD (Bartlett and O'Connor, 2007), it is important to recognize and reflect on the factors informing the arenas of citizenships that POs create for both people with AD and their carers. This paper thus examines how the factors that shape the positioning and agendas of the AD POs inform their citizenship projects. So far, research has not addressed how the varying agendas of the POs situated under diverse healthcare systems shape citizenship identities. This paper fills this gap.

Previous studies have shown how collective health-related behavior changes in line with national differences in regulatory policies and infrastructure arrangements (Jasanoff, 2011; Aarden, 2016a,b). For example, BRCA genetic testing “clients” are being configured as consumers purchasing tests in the US, and as citizens who can get state-sponsored access to testing and care in Britain (Parthasarathy, 2005). Variations in POs that represent a cultural fit to their national environments and health markets are therefore expected, even though they have been scarcely researched. But it is important to analyze the differences in order to identify the underlying factors that diversify (concepts of) citizenships across different countries. Thus, we compare three starkly distinct healthcare regimes: the USA, Germany and the UK to examine how distinct health policy contexts inform the citizenship projects of the AD POs. For this purpose, we first discuss the theoretical considerations behind studying the AD PO's role in shaping citizenship. Then we present the findings from a website and secondary literature analysis of three leading POs in three countries to elucidate how each develop distinct citizenship projects due to differences in AD policies and patient rights in their respective countries. This is concluded by a comparative section, identifying the key factors that set these citizenship projects apart.

## Theoretical Background: Role of Patient Organizations in Undertaking Citizenship Projects

Citizenship has become a key lense in disability studies, which uphold the view that politicization of the personal experience of marginalization could improve people's social situation (Campbell and Oliver, 1997; Bartlett and O'Connor, 2007). Citizenship is thus seen as a status attached to rights as well as a practice through which individuals relate to the community and the state (Prior et al., 1995). Yet the category of citizen is criticized for emphasizing cognitive knowledge (Kontos et al., 2017) that alienates for instance, people with dementia (Post, 2000). Therefore, in order to recognize how AD changes the process of constructing one's citizenship identity, it is important to recognize the different processes influencing citizenship formations.

The ways in which citizenship gets constructed through one's organizational and state affiliation is categorized by Vigoda-Gadot and Golembiewski (2004) and further developed by Baldwin and Greason (2016). They developed four arenas in which citizenship is practiced and contested. These can be used to analyze sociopolitical practices as well as related discourses: (a) Meta citizenship; (b) Macro citizenship; (c) Midi citizenship; and (d) Micro-citizenship. Meta citizenship refers to the actions taken by organizations like POs at a communal and national level to secure political changes for its members and frame people's identities. Macro-citizenship becomes the arena of individual action at communal or national level like volunteering or fundraising that ties individual to a sense of social responsibility. Midi citizenship refers to actions taken at a collective level to influence organizational practices, while Micro citizenship refers to action taken by individuals in interactions with others (Vigoda-Gadot and Golembiewski, 2004; Baldwin and Greason, 2016). In this paper, we use three of these categories developed by Vigoda-Gadot and Golembiewski (2004) and Baldwin and Greason (2016) to analyze PO's citizenship projects. However, we would like to approach these arenas of citizenship formation in context of the ‘biosocial identities’ of people, especially those living with AD (see Rabinow, 1996, p. 99).

The concept of biological citizenship was developed by Rose and Novas (2005) to understand how biosocial identities shape citizenship rights. It encompasses all other political or social citizenship projects where citizens' beliefs about their biological existence is linked to their social and political practices. Since dementia and AD are seen as causing loss of a person's ability to decide autonomously, to take political responsibility, to navigate through material and social spaces or to consent to their future care- demented person risk losing their basic citizenship rights such as dignity and autonomy (Post, 2000). Therefore, AD POs have a challenging task of enabling people to understand their changing biosocial needs. However, PO are accused of creating an idea of a univocal and stable biology that contradicts the central institution of the biosociety thesis (Lemke, 2015). Although rearticulation of somatic-oriented identities is not the exclusive domain of POs (Lemke, 2015), their advocacy, campaigns, and support services enable patients and caregivers to understand their biological conditions, rights, entitlements and enable them to claim their biological citizenship. In the disability advocacy debate, Hughes (2009) criticizes POs for creating a medicalized concept of self by ignoring the social processes of exclusion and instead politicizing the biomedical diagnosis. However, research elucidates that POs have a central role in resisting the idea of patients as passive and sick by challenging the dominant biomedical paradigm through specific biosocial claims. Studies show how POs constitute biological citizenship by producing politically wanted resources like blood, DNA, medical data etc. through biomedical research to guide the trajectory of knowledge pertaining to the disease (Novas, 2006). They also reconfigure power and knowledge by combining scientific and political discourses Valenzuela and Mayrhofer (2013) and create new forms of solidarities from shared knowledge (Gibbon and Novas, 2007). Since people with AD risk losing their ability to consent to their future care, which could compromise their dignity and autonomy, the AD POs in general and the three organizations selected for this study in particular have a voluminous task of enabling people to understand their changing biosocial conditions and secure their future medical care needs. Since their goal is to make these medicalized needs and futures recognizable, the POs adhere to a biomedicalized understanding of creating citizenship rights as opposed to a disability model.

We are aware that the ways in which people with AD can construct their citizenship claims depend upon their social situations like age, gender, ethnicity class, race etc. and its impact upon their embodied experiences (see Thomas, 1999). This paper looks at the activities of POs and their agendas to understand how geopolitical locations create different avenues for people's construction of citizenship identities. Therefore, we would not be able to analyze the impact of intersectionalities on citizenship practices at an individual level. Again, Brown et al. (2004) have suggested a 3-fold categorization for POs: (a) Support organizations for improving access to or provision of health care services; (b) constituency-based health movements addressing health inequality and inequity based on citizenship, race, ethnicity, gender, age, class and/or sexuality; and/or (c) embodied health movements addressing disease, disability or illness experience often by challenging science and practice on etiology, diagnosis, treatment, and prevention. In this paper, this categorization does not work or at best the actives of the AD POs that we study fall across the categories.

## Methodology

### Selection of POs

In order to examine how the POs in the three chosen countries—US, Germany and UK—create arenas for people with AD to practice their citizenship identities, we analyzed their websites and related secondary documents. Our aim was to understand their publicly stated agendas. We selected three national umbrella AD POs that comprise large membership and are involved in advocacy, support and research: the Alzheimer's Association in the US (ALZ) (https://www.alz.org/index.asp), the Deutsche Alzheimer Gesellschaft (German Alzheimer's Association) in Germany (DAG) (https://www.deutsche-alzheimer.de/) and Alzheimer's Society in the UK (AS) (https://www.alzheimers.org.uk/). Although the term “patient” does not appear in the names or description of any of these associations, we use the term PO as an umbrella category generally encompassing the various kinds of organizations that were established to represent and advocate the collective interests of people with AD and their carers/family.

We chose these three countries for comparison due to their starkly distinct healthcare regimes, based on the classification of healthcare systems drawn by Böhm et al. (2013). According to them, the UK healthcare system constitutes of the National Health Service (NHS) where health-care regulation, financing and service provision are funded by the State through a tax-based system. They classified the US healthcare system as a private health system, where private actors assure the regulation, financing and service provision. The German healthcare system has been classified as a social health insurance system where healthcare regulation and financing are done by the state through compulsory insurance, but the service provisions are carried out by private institutes.

While such distinctly differing systems place these countries apart, AD care has been in the mandate of the national agenda of these three countries since the past few years. In 2009 the National Alzheimer's Strategy has been published in the UK, in 2011 the National Alzheimer's Project Act (NAPA) was passed in the USA and in 2012 the Local Alliance for People with Alzheimer's was constituted in Germany, which should in 2019 result in a national strategy as announced by the German Health Minister in September 2018.

### Analysis of Web-Materials as Documents of Self-Representation

Websites were analyzed as a communication medium that create effects on the users (Bunz, 2001). Analyzing the websites of POs therefore has its merits as it provides an insight into the agenda of the website providers. It is reflective of the activities and achievements of the POs through which they aim to create avenues for people with AD and their carers to identify and claim their rights. We analyzed the websites of the three AD PO using the method of inductive content analysis (Riff et al., 2014) between March and September 2018 (see Table 1). We analyzed these websites manually by navigating through each section of their webpage. We created four units of analysis to appropriately capture the PO's role at communal and individual level in creating spaces for enacting citizenship identities. These were: (*1) the PO's relation to the healthcare system; (2) the PO's role in ensuring care; (3) the PO's involvement in the health policy; and (4) the PO's involvement in research* (see Table 1). We used these four thematic units to manually search for content in the websites. We selected passages as quotes from the websites and tabulated them under these four units. We coded them manually using open codes. We additionally referred to relevant secondary documents on these POs to substantiate the findings under each unit of analysis. Eventually we arrived at five codes under which we analyzed the activities of each PO. The findings under each code enabled us to identify how the roles of the three POs varied under each units of analysis. A close comparative look at the factors that differentiate the PO's activities from each other enabled us to identify in what ways does the national context influence how POs create arenas for people with AD and their carers to construct their meta, macro or micro citizenships.

**Table 1:** Inductive content analysis of the AD PO websites.

**PO and Country**	**Thematic Units of Analysis**	**Quotes (reworded/translated)**	**Codes**
Alzheimer's Association (ALZ), USA	The PO's relations to the healthcare system	Lobby organization	Role
		Finding suitable coverage of their care expenditures	Role
	The PO's role in ensuring care	Secure basic care for people living with AD and help them in their financial planning	Health and social improvement
		List the possible costs and detail the various financial plans and eligibilities	Care cost
		Connect them to community support services for low cost or free AD care	Support
		Offer care services on a “sliding scale”	Support, care cost
	The PO's involvement in the health policy	Diligently work(s) to make AD a national priority	Advocating change
		Better health and long-term coverage to ensure high-quality, cost-effective care	Care cost
		Urging policymakers to take historic steps for addressing the Alzheimer's crisis	Advocating change
		Our network of advocates, and we'll send you timely alerts to take simple actions that will help influence national policy and create widespread awareness of this devastating disease.	Advocating change
		Create public health infrastructure for implementing Alzheimer's interventions	Health and social improvement
	The PO's involvement in research	AD free world	Advocating change
		A breakthrough in AD treatment to attract government investment	Research strategy
		Detection of early onset of AD to reduce cost and out of pocket expenses	Care cost
		Free clinical studies	Care cost
		Don't just hope for cure. Help us find one	Research strategy
Deutsche Alzheimer Gesellschaft e. V(DAG e.v.), Germany	The PO's relations to the healthcare system	Representative at federal body	Role
		Gain social acceptance	Role
	The PO's role in ensuring care	A life with dignity and independence	Health and social improvement
		Promoting early assistance in care and financial planning before the burden of the disease becomes unmanageable	Care cost
		Improving the participation and representation	Advocating change
		Educate the young people	Advocating change
		Communicates the importance of having a supplementary insurance in accordance to expected funding gap	Care cost, advocating change
	The PO's involvement in the health policy	Ensure patient representation at the political level	Advocating change
		Representation through their drafted statements on bills and legislations	Advocating change
		Draft ethical statements	Advocating change
	The PO's involvement in research	Key focus remains on psychosocial research	Health and social improvement
		Rejects experimental procedures	Research strategy
		Urging people to be involved in the trials only when the line of treatment has proven beneficial for similar diseases.	Research strategy
		Propose to combine evaluated biomarkers and imaging techniques for reliable AD diagnosis	Research strategy
Alzheimer's Society (AS), UK	The PO's relations to the healthcare system	Part of the voluntary health care sector	Role
		Improving personalization of care	Role
	The PO's role in ensuring care	Personalization of AD care	Health and social improvement
		Maximize the effectiveness of the personal budgets	Care cost
		Training staff	Health and social improvement
	The PO's involvement in the health policy	Lobbying for AD rights through major campaigns	Advocating change
		To reach out to everyone from the time of diagnosis to offer help, and deliver a universally accessible support and advice service	Advocating change
	The PO's involvement in research	Breakthrough toward an “AD free world”	Health and social improvement
		Clinical trials and social research	Research strategy
		Research as an “opportunity to make a difference for the future”	Research strategy

## Results

### Advocating for Change: the Case of US Alzheimer's Association (ALZ)

ALZ operates on the backdrop of a free market dominated health care system (Fox and Lambertson, 2011). The lack of a formal scope for participation of patients in US healthcare delivery during the 1980's gave rise to patient advocacy groups like ALZ who protest and lobby as part of a larger political action (see Tritter and Lutfey, 2009). Due to the privatized healthcare, a primary challenge in front of its patients is finding suitable coverage of their care expenditures^1^. ALZ operates as a lobby organization to change this shortcoming. It started as an organization led by family and carers of people with AD but has transitioned towards giving representation to people with AD, especially while setting their research and advocacy agenda. Since its foundation in 1980, they have been promoting research and over the years have extended support toward financial planning for the affected. ALZ is now listed as one of the voluntary health agencies for the US under the National Health Council, which brings together diverse stakeholders within the health community to address chronic diseases and disabilities^2^.

#### Financial Planning and the Right Choice of Healthcare

Cost remains a primary concern for people with AD due to the requirement of a prolonged period of care. Given the limited health insurance coverage, ALZ offers advices on basic care and financial planning for AD^1^. They list the possible costs, financial plans and eligibilities^3^. Through their webpage, helplines, face-to-face support groups, educational sessions, online message boards and *Alzheimer's Navigator* tool for individualized care plan, they disseminate information and enable people to foresee a realistic cost^4^. They aim to supplement the healthcare deficits, minimize out of pocket expenses, make people aware of their public entitlements and connect them to community support services for low cost or free AD care^5^. By urging people to make responsible care planning through the choice of the right health insurance like the Medicaid, Madigap Insurance, Social Security Disability Insurance (SSDI) etc. that is appropriate to one's age, social, and employment status^6^, ALZ create arenas that are likely to exercise their micro-citizenships and hence actively manage their present and their biological futures.

As per the Health Care Bill under the Medicare Care Coordination Improvement Act of 2017, innovative care centers are being set up in the USA to test the possibility of offering high cost care at low cost settings^7^. But since Medicare does not cover most long-term care costs^8^. ALZ has set up local care homes as NGOs or charitable trust. They offer AD care services on “sliding scale”^9^ for payments relative to people's income. They also acknowledge the role of the caregiver and sensitize them regarding the quality of life and dignity of people with AD, especially toward the end of life. These actions are likely to expand the arenas for people to claim their micro- citizenships.

#### Political Advocacy

ALZ is the leading voice of AD advocacy in the USA and “diligently work(s) to make AD a national priority”^10^. Their advocacy slogan is “it's time to act” as they aim to secure “better health and long-term coverage to ensure high-quality, cost-effective care”^11^. They have developed the Alzheimer's impact movement (AIM), an advocacy wing to influence policy^12^. Through their website, they urge the public to become an advocate of AD and even provide online resources to facilitate the process^13^. These initiatives hold the potential of creating spaces within the public imageries and policies for recognition of the meta-citizenships of people with AD and secure their biological futures.

They aim that AD attains the status of a public health priority as the privatized healthcare system is unable to contain its costs. They are a major proponent of the National Alzheimer's Protection Act (NAPA) 2011. It granted people with AD political legitimacy^12^. ALZ's policy achievements include the *twenty first Century Cures Act*, which was legislated in order to accelerate the discovery of cures for AD. This legislation also included the *EUREKA Act* to advance research on AD. Another key achievement includes the Family Caregiver's Act 2018 that assists carers. At present, the key priority of the ALZ is the “Building Our Largest Dementia” (BOLD) Infrastructure for Alzheimer's Act to create public health infrastructure for implementing Alzheimer's interventions. These legislative victories bring AD into national limelight and hold the potential for people with AD to construct their meta-citizenships.

#### Fostering Research

ALZ's vision of an AD free world drives their research agenda. They are the largest non-profit funder of AD research worldwide. As part of their 10-year vision, they are strongly advocating for a breakthrough in AD treatment to attract government investment in research and eventually reforms. At present, they are prioritizing the detection of early onset of AD to reduce cost and out of pocket expenses. Through their research funding, peer-reviewed research (e.g., in leading journal in the field Alzheimer & Dementia), the Research Roundtable bringing together scientist of the pharmaceutical, biotechnology, imaging, and cognitive testing industry; ALZ directs the future of AD research. They claim that these roundtables involve persons affected to ensure that research really meets the need of patients and their families. Hence such spaces create arenas for the people with AD to perform their macro-citizenship rights. However, the extent to which its members set the research agenda is not known and needs further exploration.

ALZ also promotes clinical trial participation through their online matching service called *Trial Match* and lays out the safety protocols for participation^14^. They state that clinical trials entail risk but follow the same federally regulated ethical and legal codes as other medical practices. The slogan “Don't just hope for cure. Help us find one” or “Without the participation of people like you, finding a cure is nearly impossible^16^”—frames participation in trials as an “urgent help” required without which the mission for finding a cure will never be fulfilled. Thus, ALZ's strategies assigns macro-citizenship responsibilities of knowledge co-production to people with AD for the betterment of the biosocial group.

### Preserving Dignity and Rights: the Case of German Alzheimer's Association (DAG)

Patient participation and shared decision-making has become an important ideology of the German healthcare system over the past decade^15^. Hence, POs engage themselves in a wide spectrum of activities: ranging from small self-help groups to large national advocacy organizations (Geissler, 2011; Beier et al., 2016). In 2004, POs were granted the status of an observer on the Federal Joint Committee that makes important decision about health insurance benefits. All POs in Germany work under two umbrella organizations—the Bundesarbeitsgemeinschaft Selbsthilfe (the Federal Working Group of Self Help Organizations) and the Deutscher Partätischer Wohlfahrtsverbund (DPWV). They receive a small ratio of public money from the health insurances (Social Security Law V). The German Alzheimer's Association (DAG) is one of the most politically influential POs working on AD and other dementias in Germany. Founded in 1989, DAG was the first Alzheimer's organization of committed representatives. They are financed by the Federal Ministry of Foreign Affairs along with their various other funding sources^16^. Unlike the AS and ALZ, their main goal is to gain social acceptance for dementia patients and their relatives by promoting a better societal understanding of AD^17^. DAG's advisory board comprises of members with dementia, which could be seen as a significant step toward preserving dignity.

#### Developing Ways of Better Care

DAG aims toward ensuring “a life with dignity and independence” for people with AD^18^. They promote early assistance in care and financial planning for different age-groups before the burden of the disease becomes unmanageable. Through their projects on consolidating the network of contact points or “Making More Participation Available to People with Dementia,” they work toward improving the participation and representation of people with AD in health policies. A key focus of the DAG is to educate the general public especially young people and the healthcare workers^19^ and identify the gaps in knowledge and infrastructure within the present healthcare system.

Through their Alzheimer's telephone helpline, email, regional chapters and free counseling services^20^, DAG creates space for people to choose their care planning, which can enable people to construct their micro-citizenships^22^. The health insurance fund in Germany covers AD care under the long-term care services. However, certain areas of care are not covered by the compulsory long-term care funds and require an extra insurance or out of pocket expenses. Imparting such disease specific information that DAG creates an arena for people to exercise their micro-citizenships and act responsibly toward their biological futures.

#### Political Advocacy and Healthcare Initiatives

DAG's key goal is to ensure patient representation at the political level. They consider themselves a lobbyist and a deputy to the government on AD related policy decisions. Despite their observer status in the Federal Joint Committee, the extent of their influence on policy decisions is still unclear. Through their drafted statements on bills and legislations (e.g., on genetic testing, end of life care or advance research directives), they represent the voices of people with AD and their carers^21^. Some of the key bills where they suggested amendments include the Long-term Career Reform and the 4th AMG amendment. They also co-direct (with the Ministry of family affairs and health) the current alliance for a national dementia strategy.

Apart from solicited advices, DAG also critically monitors the healthcare infrastructure. They have criticized the conditions of hospital across the country, which at present are unable to provide a secondary diagnosis of AD^21^ contributing to a very late diagnosis. A unique contribution of DAG is their ethical statements through their working group “Ethics” of the German Alzheimer Society^21^. These ethical recommendations range from issues of dealing with the will of living people, to diagnosis, education or even guidelines for accompanying people in their dying phase and indirectly takes the form of political recommendations. Therefore, they attempt to ethically situate the rights, way of life and entitlements of people with AD in policies and public discourse and enable the recognition of their meta-citizenships.

#### Research Participation

DAG has problematized the participation of people with AD in biomedical research because of consent issues. They are not averse to research and provide rather small grants mainly for clinical research (e.g., research on the safety and efficacy of drugs) and care research (e.g., feasibility and efficacy of non-drug interventions)^22^. Their key focus remain on psychosocial research (in the field of Neurobiology, Gerontopsychiatrie or Nursing Science), prevention, early diagnostic as well as drug development. They have also conducted various social research projects like developing e-learning programme on dementia for relatives^23^ or opening multi-generational houses to people with dementia^24^. At present, they are focussing on improving the situation of young people with dementia and their relatives through the European RHAPSODY programme^25^. As an organization, they have issued a statement in 1999 on ethics of dementia research that rejects experimental procedures like pre-implantation diagnosis or embryonic or adult stem cell research for finding cure of the disease without knowing its cause. They argue that such procedures breach human integrity and can violate people who are no longer able to consent. They insist on research that investigates the causes of AD in order to eventually find a cure. By focusing on the role of comprehensive diagnostics for an early detection, they aim to arrest the occurrence of the disease. Therefore, DAG creates a protective arena for the performance of macro-citizenship of people with AD by urging them to participate in the trials only when the line of treatment has proven beneficial for other similar conditions. Ethicists have argued that it is the relatively less permissive German ethical and legal discourse that informs the DAG's position on research priorities and grant allocation (Schicktanz, 2015).

### Fighting for Change: the Case of Alzheimer's Society (AS) in the UK

POs in the UK are substantially involved in health policy making as part of the voluntary healthcare sector under the National Health Service (NHS) (Baggott and Jones, 2014). As per the Ministry of Health guidance and a statutory grants scheme under Section 64 of the Health Services and Public Health Act (Baggott and Jones, 2014, p. 203), the voluntary health care sector in England has a significant role to play in health service delivery. AS is one of the largest Alzheimer's charities in the UK and the NHS England lists them as a certified organization. Since POs funded by the state have been witnessing massive budget cuts, AS operates through a self-help strategy (Baggott et al., 2004). They raise funds through membership and research budgets from corporate partners, foundation and pharmaceutical companies.

#### Personalization and Participation in Care

As per the Social Care Act 2014, AD care is covered by the social support depending on the assessment carried out by the local healthcare representatives. Following the Care Act 2014, personalization i.e., increased participation of the patients in their care decisions has become a key social care agenda in the UK^26^. The care initiatives and campaign agendas of the AS reflects that, “personalization” of AD care has become their motto. As a voluntary sector partner of NHS England on its Integrated Personal Commissioning (IPC), AS is aiming to improve the coordination between officials and users in order to maximize the effectiveness of the personal budgets. A personal budget is money that a local authority allocates to a person who needs care and support in England. Scotland and Wales has a system of direct payment. In order to make personalization effective, AS trains the local authorities to bring cultural and systemic changes^27^. They urge people with AD to take control over their existing healthcare entitlements and make informed decisions about personal budgets and advanced care directives. Through its personal budget campaigns, online forums, local events, dementia cafes, and toolkit for local authorities; they create arenas for individuals to construct their “micro-citizenship” identities.

#### Political Advocacy and Initiatives

AS significantly lobbies for AD rights through major campaigns, consultations with Parliamentarians and local events. Following Alzheimer's Society Cymru's successful 45,000 reasons campaign, the Welsh Government decided to prepare the first ever Alzheimer's Strategy for Wales^28^. AS has also been a major proponent of The Mental Capacity (NI) Act 2016 and is involved in drafting its Code of Practice to be developed by the Department of Health^29^. This Act will support people with AD to make decisions about their own health, welfare and finance when they have capacity to do so.

They have also been actively involved in the implementation of the Prime Minister's Challenge on Alzheimer's launched in 2012^30^. It was followed by a second Prime Minister's Challenge of 2015 that pledged to make UK the most AD friendly country and the most suitable place for conducting AD research. Under this scheme they received 5.3 billion pounds Better Care Fund. The Dementia Friends Campaign^31^ is a direct outcome of this fund. Additionally, they have also launched a “New deal on Dementia” to make AD diagnosis available to every British citizen by 2022. As a part of this New Deal, AS aims to achieve a “New Deal on Support” to anyone in need, “New Deal on Society” by mainstreaming Alzheimer's rights and a “New Deal on Research” to find a breakthrough in research^32^. As they frame it: “Our ambition, by 2022, is to reach out to everyone from the time of diagnosis to offer help, and deliver a universally accessible support and advice service.”^33^. Such a vast initiative attracting the attention of policy-makers, scientists as well as families and friends of people with AD offers legitimacy to the care and research needs on AD and provides avenues for constructing the meta-citizenship identities of people with AD.

#### Investments in Research

AS intensively promotes biomedical research to find a breakthrough toward an “AD free world” as well as social science research to improve quality of life for people living with AD. As a part of its “New Deal on research,” they aim to make the biggest investment in Alzheimer's research by 2022^34^. They pledged 50 million pounds to set up the UK's first dedicated Alzheimer's Research Institute and have already spent 100 million pounds on pioneering research interventions. They acknowledge the pharmaceutical investments in their research initiatives but lists out an ethical protocol on their relations with commercial organizations in order to strictly regulate such links.

They have various research funding schemes and fund early career researchers. As a part of their research strategy, they lay emphasis on the involvement of patients and public in AD research, which could range from completing questionnaires to taking part in clinical trials, genetic tests or focus group discussions. AS presents research as an “opportunity to make a difference for the future” and not as a must^35^. Through such appeals they individualize people' responsibility to become active co-producers of knowledge and create arena for them to claim their macro-citizenship identities.

## Comparing the Impact of Different Healthcare Systems in Shaping PO'S Citizenship

The different healthcare regimes shape the specific care provisions and generate needs of people with AD and their carers, which the POs in each country address through their programmes. However, our comparison of the three PO's care, advocacy and research activities reflect that their key agendas and engagements with people with AD are influenced by each country's health coverage system. The nature of healthcare coverage determines the healthcare entitlements of people with AD. Although both the UK and the US have a national AD plan, and Germany is in the process of drafting one to address its challenges; the structure, nature of funding and governance of the health systems shape the ways in which these AD plans address rights and ontology of the disease. The coverage or funding structure of each healthcare regime i.e., whether it is a privatized or insurance based or public health system, shapes citizens' entitlement and sets the moral and ethical tone of the disease narrative. POs in each country thus create arenas for people with AD and their carers to understand and claim their rights under their existing healthcare provisions. The diversities in PO's activities and engagements opens varied arenas for practicing and claiming citizenships. Through their care planning, support and advocacy programmes, POs take actions toward securing political changes, run programmes to offer communal support spaces, urge individuals to make efficient care planning and contribute toward their biosocial future by engaging in research. Each of these activities open spaces for enactment of citizenships be it micro citizenship, meta citizenship or macro citizenship. However, since AD involves long-term care, the ways in which the three POs advice and enable the people with AD to plan their care expenses set apart the nature of the arenas within which their citizenships are enacted (see Table 2).

**Table 2:** Comparing the impact of different healthcare systems in shaping PO's citizenship projects.

**Health policies by countries**	**Citizenship projects**			**Impact of health policies on citizenship projects**
	**Care**	**Policy advocacy**	**Research**	
USA: Privatized healthcare system without any universal coverage for AD. NAPA overlooks AD care plan and actions. But at present, Medicare, and Medicaid covers the care of only selected population	ALZ creates arenas for people with AD and caregivers to exercise their micro citizenship by advising on financial planning of care costs, choice of healthcare options, and insurances. They also set up care homes to offer care services on a “sliding scale.” They also creates arenas for micro-citizenships through support groups, online message boards, Alzheimer's Navigator that encourages to make efficient healthcare planning to overcome the financial precarity	Through their advocacy and campaigns they frame AD as a public health priority and create arena within the healthcare policies and public imageries for recognition of the meta-citizenship of people with AD	They urge the people with AD to exercise their macro citizenship responsibilities by taking up the responsibility of contributing to research in order to find an urgent cure of AD for the collective	Cost of AD in a privatized healthcare setting alongside lack of universal coverage creates an urgency of recognition of AD as a public health priority. Thus solutions to arrest AD whose cost the healthcare setting would be unable to bear shapes ALZ's initiatives
Germany: Insurance based healthcare system that is semi-public and semi-private. AD is legislated under the Local Alliance for People with Alzheimers Act. AD care is covered by the insurance under Nursing Care Act but long-term care costs require additional insurance	DAG creates arenas for people with AD to exercise their micro citizenship by offering assistance for planning their long-term care costs, which is not covered by the Nursing insurance and requires an extra insurance. They also create arenas of micro-citizenship through their helplines, emails, regional chapters etc. that aims to enable individual to take actions within a collective space	Through their observer status in the Federal joint committee, advocacy for bills and reforms as well as critical statements on political resources on AD, they create arenas for the recognition of meta citizenship of people with AD to ethically integrate their rights within the society	They reject participation of people with AD in experimental research procedure and consider the same to be violating the integrity of people with AD. They uphold their macro-citizenship by enabling their contribution and participation in psychosocial and non-experimental biomedical research to improve lives of people with AD	The DAG‘s projects centers around bridging the gaps in AD care resulting out of a semi-privatized healthcare system that only partially recognizes the care needs. It works toward representing the actual needs of people with AD better and ensure suitable access to care and planning. Their initiatives and citizenship project thus aim toward better integration of people with AD
UK: State sponsored public healthcare system. The National Alzheimer's Act covers the care for people with AD. As per the Social Care Act 2014, Alzheimer's care is covered by the state and personal budgets are available at the local health centers under the NHS to maximize care. The Mental Health Capacity Act also uphold rights of people for a dignified death	AS creates arenas for people with AD to exercise their micro citizenship by training local authorities and people with AD about better utilization and access of personal budgets and hence improve personalization of AD care They also create arenas of micro-citizenship through their dementia cafes, virtual groups etc. to offer people knowledge about their existing rights and advance planning for end of life decisions	Through their campaigns and political engagements, they attempt to uphold AD as a healthcare priority and frame people with AD as meta citizens whose biosocial needs require recognition within the existing public health setting for increased integration and healthcare expenditures	They uphold macro-citizenship by encouraging people with AD and their carers to take part in research as an opportunity for individual action that will bring a solution for the entire biosocial community	Identifies gaps in the public healthcare provision and the universal healthcare rights to ensure better utilization of care services and budgets. Personalization and improving the efficiency of existing care and research shapes the initiatives of the AS and their citizenship projects

In the US, the ALZ focuses on disseminating information on various insurance coverages in order to enable individuals choose and manage their care costs efficiently. They advocate for AD to be seen not just as a disease but as a cause' in order to attract political attention towards the unmanageable expenses of people with AD and their carers. They frame AD as a public health priority and create arenas for recognition of meta-citizenships of people with AD. Prompted by the privatized healthcare system that is still unable to provide AD coverage despite the provisions of the national AD plan, ALZ attempts to enhance the arenas of micro-citizenship by encouraging people to identify suitable insurance or Medicare/Medicaid entitlements. They also fulfill a key care deficit as part of their care programme through their local affordable care home projects. The information disseminated by them has a special focus on managing the cost of care and advocating policy changes toward a future where AD costs are affordable.

ALZ's framing of AD as a public health priority, holds the potential of creating an arena within the healthcare policies and public imageries for recognition of the meta-citizenship of people with AD. Their public engagement activities and awareness drives also work toward bringing in major legislative changes and public health investments. The neo-liberal healthcare infrastructure, overall healthcare deficits and lack of public care initiatives result in the ALZ directly urging its patients to participate in clinical trials as a responsibility toward their biosocial community to bring urgent breakthrough in research. Thus, ALZ's programmes focus on creating economic sustenance and constructs the spaces for performance of citizenships at various levels- micro, macro, or meta.

In Germany, the burden of responsibility upon the patients to claim their rights is not as strong as in the US because the private insurances under the Nursing Care Act covers the care costs of people with AD. However, DAG keeps the patients and relatives informed about the legal and financial necessities of planning their long-term care expenses as the insurance system does not cover long-term AD care costs. They take social initiatives like multi-generational houses to create awareness among the teen-agers and bridge gaps within the existing social care system. Such initiatives can create arenas for people to identify their micro-citizenships. Their observer status in the Federal joint committee, advocacy for bills and reforms as well as critical statements on political resources on AD hold the potential to create arenas for the recognition of meta citizenship of people with AD. Given the importance assigned to personhood within the German ethical discourse due to the historicity of Nazi Medicine where psychiatric patients including patients with dementia were killed in so called euthanasia programme (Schicktanz, 2017), DAG's activities focus on making the people with AD and the larger society aware of the dignity and respect that they deserve. Hence, they do not find it appropriate to involve patients in experimental research, who are unable to consent, especially since AD has no proven cause and its results have no direct benefit for the participants. Thus, they create conditional grounds upon which people with AD claim their macro-citizenship rights. These historical forces along with a semi-privatized insurance-based health system that falls short of recognizing specific care needs of people with AD, prompts DAG's efforts toward better integration and recognition of citizenship rights of this biosocial group.

In the UK, the AS aim to ensure patient's “choice and control over their own care and support” through their “Personal Choice Programme”^36^. Personalization has become an important part of the British healthcare agenda so that the patients become aware of their person-specific needs and make active claims over it. They create arenas for people with AD to exercise their “micro citizenship” by training local authorities and people with AD about better utilization and access of personal budgets. Through their dementia cafes, virtual groups etc. they educate people about their existing rights and advance planning for end of life decisions. These can potentially create avenues for people to assume their individualized responsibilities as micro-citizens. Basic recognition of the disease is key to attract public spending in AD care and research, Hence, through their campaigns and political engagements, they attempt to uphold AD as a healthcare priority and frame people with AD as meta citizens whose biosocial needs require recognition within the existing public health setting. Their research agenda focuses on early detection that stems from ensuring individualized care needs and reducing the overall cost on the public healthcare system. By encouraging people with AD and their carers to take part in research as an opportunity for individual action that will bring a solution for the entire biosocial community, they create avenues for them to uphold their macro-citizenship responsibilities. Thus, ALZ create arenas for citizenship performances- be it micro, meta or macro by urging people with AD to be choice-centric and attempting to better integrate their needs within the current public healthcare system.

Although the AD POs in most western countries resist stigma and exclusion of their patients' and caregivers' and strive to get AD recognized as a healthcare priority, we know that the AD landscape varies between countries and so does the focus of the AD POs. In order to accommodate the national differences, the Alzheimer's Disease International (ADI), which is the international federation of Alzheimer's associations, work toward fighting AD globally by finding the solutions locally. Alzheimer Europe, the consortium under the European Parliament also works to ensure that AD receives a priority at the European level through national solutions. In order to find these national level solutions for factoring in the needs of people with AD at an international level, it is important to understand how distinct national healthcare systems support people's biosocial needs and inform their citizenship experiences at the micro, meta, or macro levels.

## Conclusion

This paper contributes toward enhancing our understanding of the role of healthcare systems in the PO's agendas and their arenas for citizenship projects. Our findings indicate that since AD involves long-term care, which is expensive, the ways in which the three POs enable the people with AD to plan their care expenses set apart the nature of citizenship enactments. Thus, clearly, the way in which POs create arenas of macro, micro and meta citizenship differ based on how AD care and research is addressed by the respective healthcare systems. From the perspective of organizational sociology, this finding is in line with the neo-institutional approach in the context of “path dependency,” which implies institutional persistence and even fixation (Acheson, 2014) that follows historical-contextual rules and architectures (Bevir and Rhodes, 2010). Such path-dependency is also functional, representing “cultural fit,” and not (as often seen by neo-institutionalism) a constraint to change due to embeddedness in founding conditions, values, knowledge and structures (Romanelli, 1991; Ramanath, 2009). Our comparison of the activities of ALZ, DAG, and AS through their websites reflect that their activities foster differential modes of performing or contesting citizenships that are in line with their national socio-ethical context. The POs' agendas are shaped in response to the respective health policies on AD, which constructs the disease narrative. Our analysis shows that the way each of the POs work toward enabling its members to contest the existing disease narrative within the respective healthcare regime depends upon how the health systems are funded and allocate resources for AD care.

However, we recognize that this paper has a methodological drawback as it relies mostly on website analysis. Websites contain proclamatory statements and we do not know how correctly they reflect the reality. But of course, the ideologies and statements are important leads and future research can examine the social realities behind them. Furthermore, we concentrate on the largest and most politically influencing POs in the three respective countries to make the comparison more consistent. By this, we do not cover the whole spectrum of POs existing within these countries. As former studies indicate, some smaller POs have stronger inclusion policies for persons with dementia than these three (Schicktanz et al., 2018). Future course of research could empirically investigate a spectrum of AD POs spread across two or more countries, holding comparative relevance in terms of having similar socio-cultural and economic infrastructure but differing or similar healthcare regimes. Such a research could capture how people with AD and their carers identify as political citizens and biological citizens through their association with the AD POs.

## Data Availability Statement

All datasets generated and analyzed for this study are cited in the article/supplementary material.

## Author Contributions

SM conceptualized, carried out the background research, and drafted the manuscript. SS helped in the conceptualization of the paper, offered background insights and theoretical inputs, commented, and worked on various drafts of the paper.

### Conflict of Interest

SM and SS declare that the submitted work was not carried out in the presence of any personal, professional or financial relationships that could potentially be construed as a conflict of interest.

## Footnotes

^1^https://www.alz.org/care/alzheimers-dementia-costs-paying-for-care.asp (accessed March 12, 2018).

^2^http://www.nationalhealthcouncil.org/about-nhc/members/alzheimers-association (accessed March 9, 2018).

^3^https://www.alz.org/care/alzheimers-dementia-common-costs.asp; https://www.alz.org/national/documents/brochure_moneymatters.pdf

^4^https://www.alz.org/care/alzheimers-dementia-online-tools.asp (accessed March 12, 2018).

^5^https://www.communityresourcefinder.org/ (accessed March 12, 2018).

^6^https://www.alz.org/care/alzheimers-dementia-insurance.asp (accessed March 12, 2018).

^7^https://www.alz.org/living_with_alzheimers_healthcare_reform.asp#medicare (accessed March 12, 2018).

^8^https://www.alz.org/help-support/caregiving/financial-legal-planning/medicare (accessed March 12, 2018).

^9^https://www.alz.org/help-support/caregiving/care-options/adult-day-centers (accessed March 12, 2018).

^10^https://www.alz.org/about_us_about_us_.asp (accessed March 12, 2018).

^11^https://www.alz.org/advocacy/federal-priorities.asp (accessed March 12, 2018).

^12^https://alzimpact.org/ (accessed March 16, 2018).

^13^https://www.alz.org/get-involved-now/advocate (accessed March 16, 2018).

^14^https://www.alz.org/research/clinical_trials/find_clinical_trials_trialmatch.asp (accessed March 12, 2018).

^15^In 2004, the Federal Government appointed the “Commissioner for Patients of the Federal Government” to represent patient interest www.patientenbeauftragte.de.

^16^https://www.deutsche-alzheimer.de/ueber-uns/projekte.html (accessed March 12, 2018).

^17^https://www.deutsche-alzheimer.de/ueber-uns.html (accessed March 12, 2018).

^18^https://www.deutsche-alzheimer.de/angehoerige.html (accessed March 16, 2018).

^19^http://www.alzheimerandyou.de/ (accessed March 16, 2018).

^20^https://www.deutsche-alzheimer.de/unser-service.html (accessed March 16, 2018).

^21^https://www.deutsche-alzheimer.de/ueber-uns/stellungnahmen.html (accessed March 16, 2018).

^22^https://www.deutsche-alzheimer.de/die-krankheit/forschung/aktuelle-forschungsaufrufe.html (accessed March 13, 2018).

^23^https://www.deutsche-alzheimer.de/ueber-uns/projekte/neue-wege-in-der-angehoerigenunterstuetzung.html (accessed November 1, 2018).

^24^https://www.deutsche-alzheimer.de/ueber-uns/projekte/mehrgenerationenhaeuser-fuer-menschen-mit-demenz.html (accessed November 1, 2018).

^25^https://www.deutsche-alzheimer.de/ueber-uns/aktuelles/artikelansicht/artikel/forschungsprojekt-rhapsody-schickt-neuen-online-ratgeber-zu-demenz-im-juengeren-lebensalter-in-den-te.html (accessed March 16, 2018).

^26^https://www.alzheimers.org.uk/info/20091/what_we_think/207/personalisation (accessed March 17, 2018).

^27^https://www.alzheimers.org.uk/info/20023/our_dementia_programmes/260/personal_choice_programme (accessed March 18, 2018).

^28^https://www.alzheimers.org.uk/info/20226/dementia_strategy_for_wales (accessed March 18, 2018).

^29^https://www.alzheimers.org.uk/info/20032/legal_and_financial/127/mental_capacity_act (accessed March 18, 2018).

^30^https://www.alzheimers.org.uk/info/20090/national_policies/46/prime_minister_s_challenge (accessed March 18, 2018).

^31^https://www.alzheimers.org.uk/info/20002/get_involved/1071/dementia_friends (accessed March 18, 2018).

^32^https://www.alzheimers.org.uk/sites/default/files/migrate/downloads/alzheimers_society_2017-2022_the_new_deal_on_dementia.pdf (accessed March 18, 2018).

^33^https://www.alzheimers.org.uk/download/downloads/id/3384/alzheimers_society_2017-2022_the_new_deal_on_dementia.pdf (accessed March 18, 2018).

^34^https://www.alzheimers.org.uk/info/20024/strategy_and_annual_reports/326/our_strategy_2017-2022 (accessed March 18, 2018).

^35^https://www.alzheimers.org.uk/info/20020/take_part_in_research (accessed March 18, 2018).

^36^https://www.alzheimers.org.uk/about-us/our-dementia-programmes/personal-choice-programme (accessed March 18, 2018).
